# Jackhammer Esophagus Associated with a Pulmonary Vein Antrum Isolation of Atrial Fibrillation

**DOI:** 10.31662/jmaj.2021-0028

**Published:** 2021-07-06

**Authors:** Togo Sakai, Masao Takemoto, Eiji Nyuta, Takuya Tsuchihashi

**Affiliations:** 1 Cardiovascular Center, Steel Memorial Yawata Hospital, Kitakyusyu, Japan

**Keywords:** Atrial fibrillation, Jackhammer esophagus, Pulmonary vein antrum isolation, Radiofrequency catheter ablation

Jackhammer esophagus (JE) is a hypercontractile esophageal disorder. Peristalsis is preserved and distal contractile latency is normal in JE, which differentiates it from achalasia and distal esophageal spasm, respectively ^(1)^. A 68-year-old man was admitted to our hospital with a chief complaint of palpitations due to drug-refractory atrial fibrillation (AF) to undergo pulmonary vein antrum isolation (PVAI) and box isolation of the posterior wall of the left atrium. The day after PVAI of AF was performed, he experienced difficulty in swallowing meals and vomited. Three-dimensional computed tomography revealed that his esophagus was tapered and became hypercontractile (arrow in Figure 1). PVAI can rarely provoke esophageal and nerve damage due to thermal injury ^(2)^. Thus, the damage might affect esophageal vagal nerve stimulation, resulting in esophageal hypercontractions ^(3)^. Finally, he was diagnosed with JE. He had fasted for 2 days and then started tender meals. Nine days after undergoing PVAI, he demonstrated complete recovery. He has remained well without any symptoms for 6 months after undergoing PVAI. To the best of our knowledge, this is the first reported case of JE associated with PVAI of AF in Japan.

**Figure 1.**
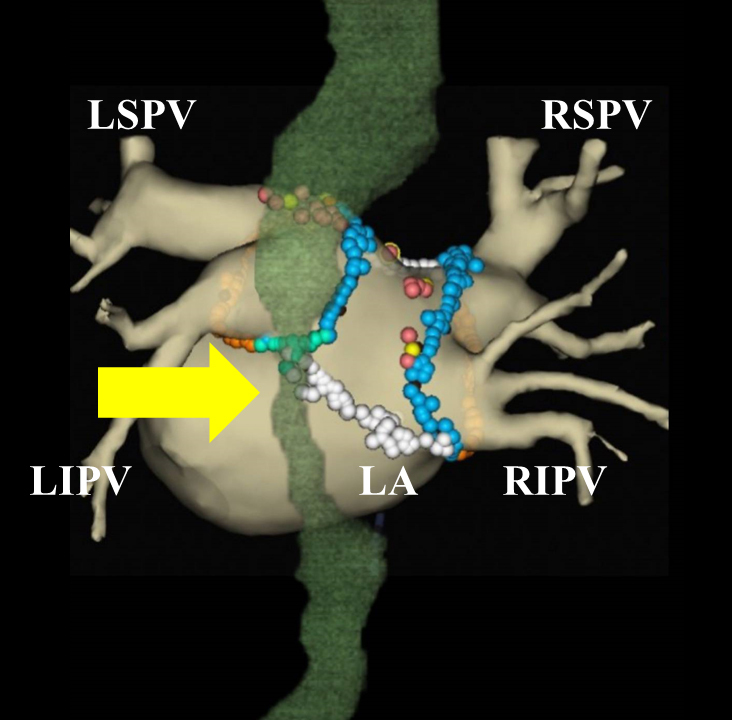
Three-dimensional computed tomography revealed that the patient’s esophagus (green) was tapered and became hypercontractile like a jackhammer (yellow arrows). The colored tags indicate ablation lines. LA = left atrium, LSPV = left superior pulmonary vein, LAPV = left inferior pulmonary vein, RSPV = right superior pulmonary vein, RIPV = right inferior pulmonary vein.

## Article Information

### Conflicts of Interest

None

### Author Contributions

All authors were in charge of the patients.

### Informed Consent

Written consent has been obtained from the patient.
